# Spatial stratified heterogeneity of mumps incidence in China: a Geodetector-based analysis of driving factors

**DOI:** 10.3389/fpubh.2025.1637288

**Published:** 2025-08-12

**Authors:** Ke Hu, Chaojie Li, Xingjin Yang, Di Xiao, Xing Zhang, Mingyang Yu

**Affiliations:** ^1^Xiamen Haicang Hospital, Xiamen, Fujian, China; ^2^Xingtai Center for Disease Control and Prevention, Xingtai, Hebei, China; ^3^QianDongNanZhou Center for Disease Control and Prevention, QianDongNanZhou, Guizhou, China; ^4^Community Health Service Center of Jiuxian Tongliang District, Chongqing, China; ^5^Nanjing Lishui Dongping Street Health Center, Nanjing, Jiangsu, China; ^6^Fuwai Central China Cardiovascular Hospital, Zhengzhou, Henan, China

**Keywords:** mumps incidence, spatial autocorrelation, spatial stratified heterogeneity, Geodetector, multifactorial interactions

## Abstract

**Background:**

China reports the highest number of mumps cases globally, with the disease demonstrating distinct spatial clustering and variability characteristics.

**Methods:**

This study employed descriptive statistics and spatial autocorrelation analysis to examine the spatial distribution characteristic and patterns of mumps across 31 Chinese provinces in 2020. Furthermore, based on the principle of spatial stratified heterogeneity, the Geodetector method was systematically applied to assess the influence intensity and interaction effects of economic development, population structure, education level, environmental conditions, and healthcare resource allocation on mumps incidence rate.

**Results:**

The findings revealed a distinct west-to-east decreasing trend of mumps incidence in China, demonstrating significant spatial autocorrelation primarily manifested as high-high clustering in western areas and low-low clustering in eastern regions. Among all influencing factors, the child dependency ratio showed the strongest association with mumps incidence, while healthcare resources exhibited relatively weaker effects. Notably, significant synergistic effects were observed among risk factors, with particularly prominent interaction between GDP per capita and illiteracy rate.

**Conclusion:**

This study provides critical evidence for region-specific mumps prevention strategies, highlighting the need for integrated control measures that consider multifactorial interactions to effectively contain mumps in China.

## 1 Introduction

Mumps, an acute respiratory infectious disease caused by the mumps virus, has experienced frequent outbreaks worldwide in recent years, even in countries with high vaccination coverage (1). China, reporting the highest number of mumps cases globally (2), faces a particularly severe epidemic situation. Epidemiological data indicated that the average annual incidence rate in mainland China from 2004 to 2018 was 21.44 per 100,000 population, with significant spatial clustering (3). High-risk areas were concentrated in southern provinces such as Hunan, Hubei, Chongqing, Guizhou, Guangdong, and Guangxi (4). Despite the implementation of a two-dose mumps-containing vaccination program in China, breakthrough infections among children and young adults continued to occur (5), suggesting that socioeconomic and environmental factors, in addition to immunological factors, may play a crucial role in disease transmission.

Existing research demonstrated that mumps incidence were influenced by multidimensional determinants. First, climatic factors such as temperature and relative humidity exhibited a significant positive correlation with mumps incidence (6). Beyond environmental drivers, socioeconomic indicators—including GDP per capita and disposable income—may shape disease control efficacy by modulating healthcare resource allocation (7). Consistent with this, studies indicated that public health funding was strategically directed toward high-incidence regions to mitigate outbreaks (4). Additionally, urbanization influenced transmission patterns by altering population density (8), while education levels indirectly affected mumps risk by shaping vaccination decisions and health behaviors (9). Notably, elevated PM_2.5_ concentrations had been linked to increased mumps incidence (4), and demographic factors such as household size and child dependency ratio showed significant associations with outbreak risks (9, 10).

However, current studies exhibit three major limitations: Firstly, most analyses have focused on individual provinces, lacking a comprehensive nationwide assessment of multifactorial interactions. Secondly, there has been insufficient attention to the allocation of community health services, including indicators such as the number of community health centers per 100,000 population and number of general practitioners per 10,000 population. Thirdly, conventional statistical approaches are limited in capturing nonlinear effects and spatial stratified heterogeneity across determinants. The Geodetector method offers an effective solution for quantifying both the explanatory power of individual factors and their interaction effects, making it particularly valuable for investigating the spatial differentiation mechanisms underlying health issues (11).

Therefore, this study employed the Geodetector method to systematically examine the influence intensity and interaction patterns of economic development, population structure, education level, environmental conditions, and healthcare resource on the spatial distribution of mumps incidence in China. The findings will provide scientific evidence for formulating region-specific prevention and control strategies.

## 2 Methods

### 2.1 Data

Based on existing literature and data availability, we selected 12 indicators for the year 2020, with detailed information provided in Table 1. This study was conducted at the provincial level, encompassing 31 administrative regions (excluding Hong Kong, Macao, and Taiwan). The mumps incidence data were obtained from the Public Health Science Data Center of the Chinese Center for Disease Control and Prevention.^1^ PM_2.5_ concentration data were extracted from provincial environmental status bulletins, healthcare resources data were derived from the China Health Statistical Yearbook, and all other socioeconomic data were collected from the China Statistical Yearbook.

**Table 1:** Key factors selected for analysis.

Categories	Factors	Variable symbol
Economic Development	GDP per capita	X_1_
	Disposable income per capita	X_2_
	Urbanization rate	X_3_
Population Structure	Average household size	X_4_
	Child dependency ratio	X_5_
Environment condition	PM_2.5_	X_6_
Education level	Illiteracy rate	X_7_
	Years of schooling	X_8_
Healthcare Resources	Total health expenditure per capita	X_9_
	Number of licensed physicians per 1,000 population	x_10_
	Number of community health centers per 100,000 population	X_11_
	Number of general practitioners per 10,000 population	X_12_

### 2.2 Descriptive analyses

The geographical distribution characteristics of mumps cases among the 31 districts were visually represented through thematic mapping techniques.

### 2.3 Spatial autocorrelation analysis

Spatial autocorrelation analysis is a fundamental method in geospatial statistics, primarily involving three core metrics: the Global Moran’s I index, Local Moran’s I index, and Getis-Ord Gi* statistic.

#### 2.3.1 Global Moran’s I

The Global Moran’s I index measures the overall spatial autocorrelation across the study area. Its mathematical formulation is as follows (Equation 1) (12):


(1) $$I=\frac{n\sum_{i=1}^{n}\sum_{j=1}^{n}W_{\mathrm{ij}}(x_{i}-\bar{x})(x_{j}-\bar{x})}{\sum_{i=1}^{n}\sum_{j=1}^{n}W_{\mathrm{ij}}\sum_{i=1}^{n}{\left(x_{i}-\bar{x}\right)}^{2}}$$

The Global Moran’s I index ranges from −1 to 1, where *n* represents the sample size, *W*_ij_ denotes the elements of the spatial weight matrix, *x*_i_ and *x*_j_ are the observed values, and $\bar{X}$ is the mean value. Values between 0 and 1 indicate significant positive spatial autocorrelation (clustering of similar values), values between −1 and 0 demonstrate significant negative spatial autocorrelation (clustering of dissimilar values), while a value of 0 suggests a random spatial distribution, with statistical significance determined by the standardized Z-score (13) (Equation 2).


(2) $$Z=\frac{I-E(I)}{\sqrt{\mathrm{var}(I)}}$$

Here, E(I) indicates Moran’s coefficient’s expected value and var.(I) denotes its variance. Spatial aggregation of mumps incidence is indicated when, at the 0.05 significance level, Z > 1.96 (i.e., when Moran’s coefficient has a *p*-value <0.05).

#### 2.3.2 Local indicators of spatial association

LISA was employed to identify local spatial heterogeneity patterns, calculated as (Equation 3):


(3) $$I_{i}=\frac{n(x_{i}-\bar{x})\sum_{j=1}^{n}w_{\mathrm{ij}}(n_{j}-\bar{n})}{\sum_{j=1}^{n}{\left(x_{j}-\bar{x}\right)}^{2}}$$

Using the same variable definitions as Global Moran’s I, LISA identifies four spatial patterns: high-high clusters (HH), low-low clusters (LL), high-low outliers (HL), and low-high outliers (LH) (14). These statistically significant patterns are visualized through LISA cluster maps (15).

#### 2.3.3 Getis-Ord Gi* hotspot analysis

To further identify statistically significant hotspots and coldspots of mumps incidence, we performed Getis-Ord Gi* analysis. This method calculates a Z-score for each spatial unit by comparing its value with neighboring units defined by the same spatial weight matrix used in Moran’s I analysis. Areas with Z > 1.96 (*p* < 0.05) were classified as significant hotspots (high-value clustering), while Z < −1.96 (*p* < 0.05) indicated coldspots (low-value clustering). The results were visualized in Gi* cluster maps, complementing the LISA analysis by emphasizing the intensity of spatial concentration patterns.

### 2.4 Geodetector

The Geodetector is a statistical analysis method based on the principle of spatial stratified heterogeneity, specifically designed to identify spatial differentiation characteristics and driving mechanisms of geographical phenomena. This method systematically explains spatial differentiation by quantifying the explanatory power (PD-value) of various influencing factors on spatial variation and analyzing interactions between multiple factors (16). The Geodetector comprises four components: factor detector, risk detector, ecological detector, and interaction detector (17–19).

#### 2.4.1 Factor detector

The factor detector serves as the core analytical module in the Geodetector method. Based on the spatial stratified heterogeneity theory, this method calculates the PD statistic (also called q-value) to quantify the independent explanatory power of each influencing factor (Equation 4) (20):


(4) $$\mathrm{PD}=1-\frac{\sum_{h=1}^{L}N_{h}\sigma_{h}^{2}}{N\sigma^{2}}$$

The statistical parameters include *N*_h_ (sample size of stratum *h*), *σ*_h_^2^ (variance of stratum *h*), *N* (total sample size), and *σ*^2^ (total variance), with the PD value ranging between 0 and 1, where higher values indicate greater explanatory power of the factor for spatial differentiation.

#### 2.4.2 Risk detector

The Risk Detector is primarily employed to identify and evaluate differences in risk levels across various stratified influencing factors. This method quantifies mean differences between strata using independent samples *t*-tests (Equation 5) (17).


(5) $$t_{{\bar{y}}_{h-1}-{\bar{y}}_{h-2}}=\frac{{\bar{Y}}_{h=1}-{\bar{Y}}_{h=2}}{{\left[\frac{\mathrm{Var}({\bar{Y}}_{h=1})}{n_{h=1}}+\frac{\mathrm{Var}({\bar{Y}}_{h=2})}{n_{h=2}}\right]}^{1/2}}$$

Where ${\bar{Y}}_{h}$ represents the average incidence rate of layer h, $n_{h}$ is samples, Var represents sample variance, t follows the Student’s-t-test distribution.

The null hypothesis as follows Equation 6:


(6) $$H_{0}:{\bar{Y}}_{h=1}={\bar{Y}}_{h=2}$$

If the null hypothesis is rejected at significance level *α*, we conclude that there is a significant difference in the average mumps incidence rate between the two regions.

#### 2.4.3 Ecological detector

The ecological detector is an ANOVA-based statistical method designed to assess significant differences in explanatory power between distinct influencing factors on spatial differentiation patterns of geographical phenomena. This method operates on the principle of F-statistic calculation to evaluate the significance of within-strata variance differences for two influencing factors (Equations 7–9).


(7) $$F=\frac{n_{X1}(n_{x2}-1)\mathrm{SS}W_{X1}}{n_{X2}(n_{x1}-1)\mathrm{SS}W_{X2}}$$

(8) $$\mathrm{SS}W_{X1}=\sum_{h=1}^{L1}N_{h}\sigma_{h}^{2}$$

(9) $$\mathrm{SS}W_{X2}=\sum_{h=1}^{L2}N_{h}\sigma_{h}^{2}$$

Where *n*_x1_ and *n*_x2_ represent the samples of two factors x_1_ and x_2_, respectively. *SSW*_X1_ and *SSW*_X2_ represent the sum of the within-strata variance of x_1_ and x_2_, respectively; *L_1_* and *L_2_* represent the number of layers of x_1_ and x_2_, respectively.

The null hypothesis states that both factors equally explain the spatial variation. If the calculated *F*-value exceeds the critical threshold, we reject the null hypothesis, indicating statistically significant differences in explanatory power between the factors.

#### 2.4.4 Interaction detector

The Interaction Detector quantitatively assesses interaction types between paired influencing factors and their synergistic effects on spatial differentiation of dependent variables. By comparing single-factor PD-value with interaction PD-value, this module systematically identifies five characteristic interaction patterns (Table 2) (21): nonlinear-weakening, univariate-weakening, bivariate enhancement, independent, and nonlinear-enhancement effects.

**Table 2:** Interaction types between variables.

Description	Interaction
$\mathrm{PD}(X_{1}\cap X_{2})<\mathrm{Min}(\mathrm{PD}(X_{1}),\mathrm{PD}(X_{2}))$	Weaken, nonlinear
Min(PD(X1),PD(X2))<PD(X1∩X2)<Max(PD(X1)),PD(X2))	Weaken, univariate
$\mathrm{PD}(X_{1}\cap X_{2})>\mathrm{Max}(\mathrm{PD}(X_{1}),\mathrm{PD}(X_{2}))$	Enhanced, bivariate
$\mathrm{PD}(X_{1}\cap X_{2})=\mathrm{PD}(X_{1})+\mathrm{PD}(X_{2})$	Independent
$\mathrm{PD}(X_{1}\cap X_{2})>\mathrm{PD}(X_{1})+\mathrm{PD}(X_{2})$	Enhance, nonlinear

#### 2.4.5 Categorization methods for independent variables

Continuous variables in Geodetector analysis require discretization. Table 3 summarizes the five evaluated methods with their mathematical properties and applications.

**Table 3:** Discretization methods for Geodetector analysis.

Methods	Mathematical principle	Optimal data distribution	Advantage
Natural breaks	Maximizes inter-class variance	Any distribution	Adapts to data clusters
Quantile	Equal sample size per category	Non-uniform	Robust to outliers
Equal interval	Fixed value range intervals	Uniform	Simple interpretation
Geometric interval	Geometric progression boundaries	Exponential	Handles skewed data
Standard deviation	Multiples of standard deviation from mean	Normal	Statistical significance framing

The optimal method was selected by systematically comparing all approaches across 3–8 classification levels, choosing the combination maximizing the PD value (22). This data-driven strategy ensures objective identification of the most effective discretization for each variable.

### 2.5 Software implementation

The spatial analysis workflow was implemented using the following tools and resources: Data preprocessing (including variable discretization) and spatial autocorrelation analysis were performed using ArcGIS 10.2 (ESRI, Redlands, CA). Geodetector modeling was conducted using the Excel-based software available at http://Geodetector.cn/Download.html. Base maps were obtained from the National Geographic Information Public Service Platform (approval number: GS[2024]0650). Statistical analyses employed two-tailed tests with a significance threshold of *α* = 0.05.

## 3 Results

### 3.1 Spatial distribution characteristic and aggregation characteristics of mumps incidence

**Figure 1:**
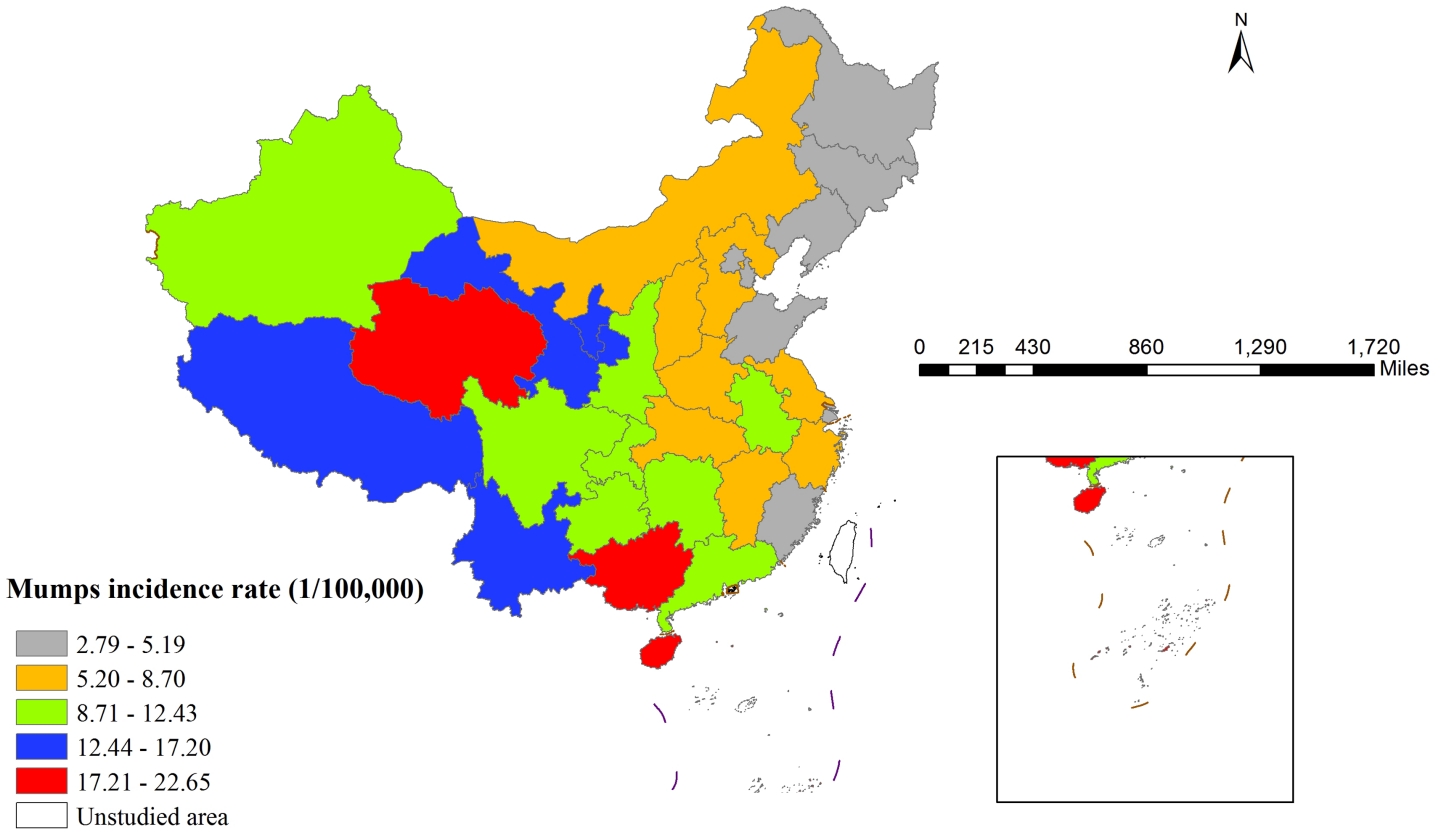
Spatial distribution of Mumps incidence rate.

In 2020, China demonstrated pronounced spatial heterogeneity in mumps incidence rates across provinces, exhibiting a distinct west-to-east decreasing gradient (Figure 1). Qinghai province recorded the highest incidence rate (22.65 per 100,000 population), whereas Heilongjiang province showed the lowest rate (2.79 per 100,000 population). This geographical distribution pattern likely reflected regional variations in socioeconomic development, vaccination coverage, and population density.

**Figure 2:**
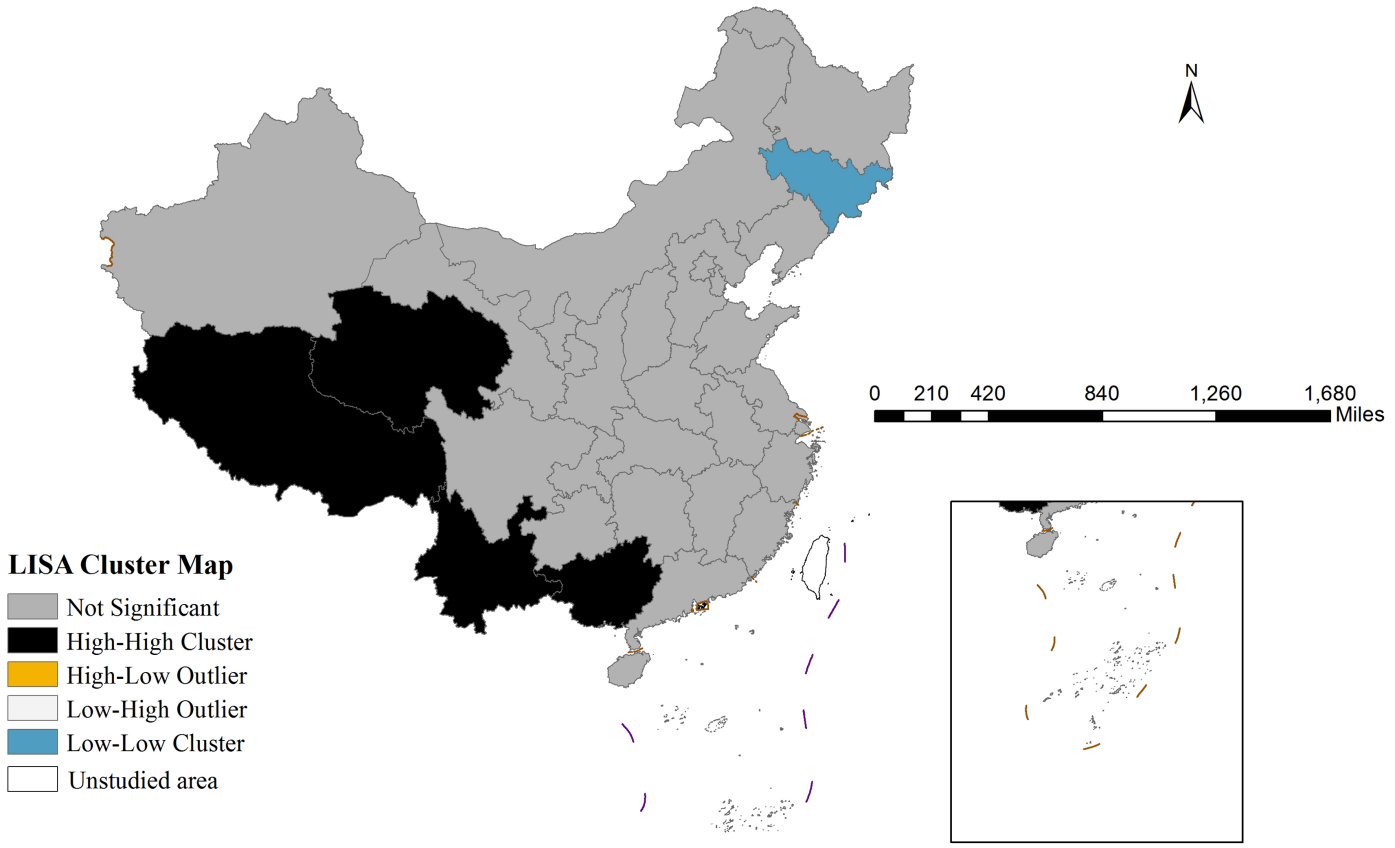
Lisa cluster map of Mumps incidence rate in China in 2020.

Global spatial autocorrelation analysis revealed a significant Moran’s I index of 0.399 (*p* < 0.001), confirming strong spatial dependence of mumps transmission. The LISA cluster map (Figure 2) further identified two predominant spatial aggregation patterns: high-high clusters were primarily concentrated in western China (Qinghai, Tibet, Yunnan, and Guangxi), that may be associated with local environmental conditions, socioeconomic factors, and population behaviors; in contrast, low-low clusters were predominantly distributed in northeastern China (particularly Jilin province), exhibiting substantially lower incidence rates that potentially indicate successful disease control measures or the influence of unidentified protective factors.

**Figure 3:**
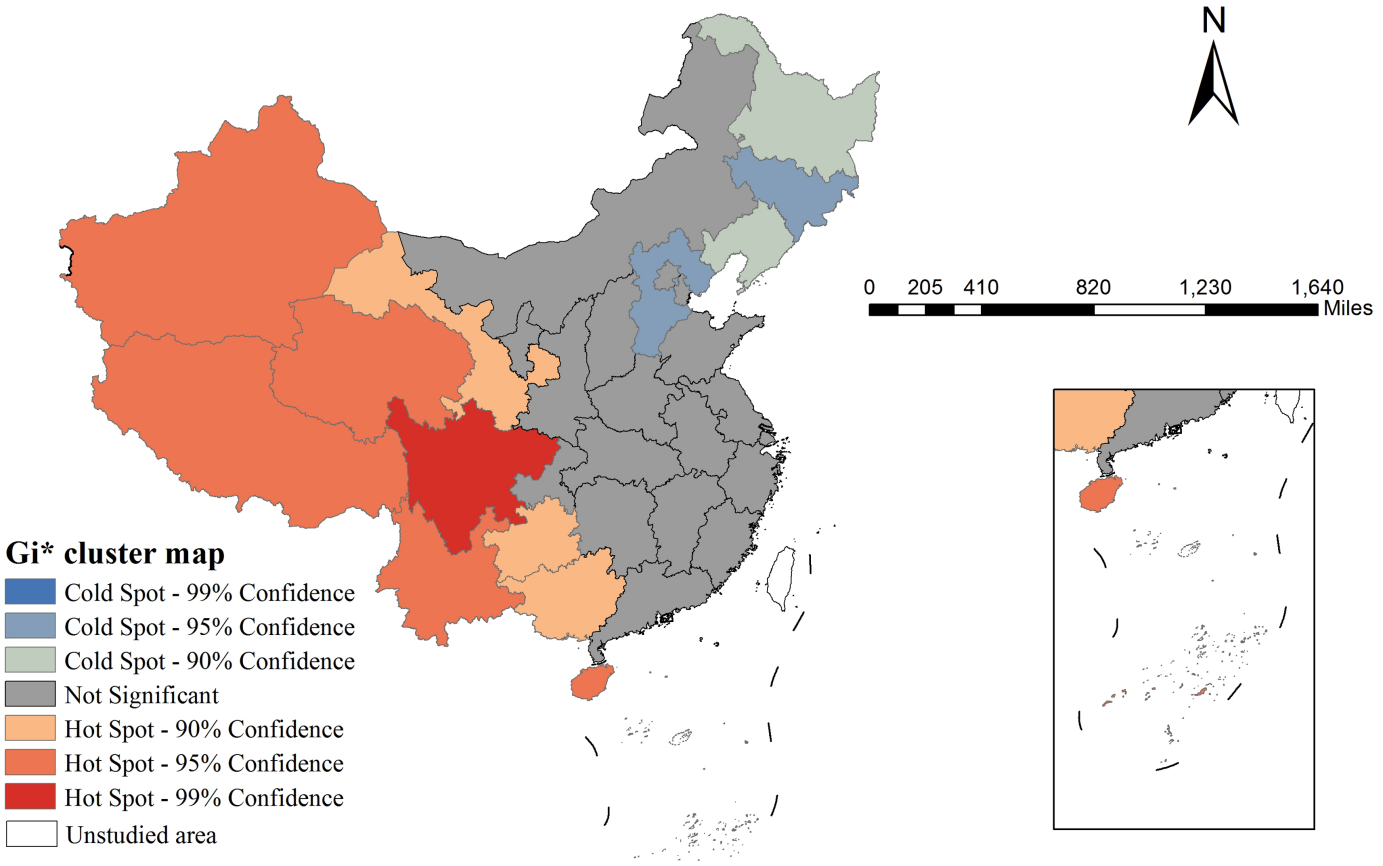
Gi* cluster map of Mumps incidence rate in China in 2020.

The Gi* cluster map revealed distinct spatial patterns of mumps incidence. Significant hotspots (high-incidence clusters) were observed in western area, showing 90–99% confidence levels. Conversely, coldspots (low-incidence clusters) were concentrated in Northeastern region with similar confidence levels (Figure 3).

### 3.2 Results of Geodetector analysis

#### 3.2.1 Variable discretization

The 12 independent variables were categorized using five distinct classification methods (equal interval, natural breaks, quantile, geometric interval, and standard deviation) with category numbers ranging from 3 to 8. For each variable, we calculated the Power of Determinant (PD) values through factor detector analysis, where PD quantifies the strength of association between an independent variable and mumps incidence (with values ranging from 0 to 1, higher values indicating stronger explanatory power). The optimal classification scheme for each variable was selected based on the maximum PD value, as detailed in Table 4.

**Table 4:** Results of factor detection and variable classification.

Factors	PD	*p*	Classification method	Classification interval
GDP per capita	0.47	0.014	Quantile	5
Disposable income per capita	0.42	0.020	Quantile	5
Urbanization rate	0.39	0.037	Quantile	5
Average household size	0.45	0.002	Natural Breaks	3
Child dependency ratio	0.54	0.031	Quantile	7
PM_2.5_	0.36	0.011	Geometrical interval	3
Illiteracy rate	0.49	0.027	Quantile	6
Years of schooling	0.48	0.007	Quantile	5
Total health expenditure per capita	0.29	0.667	Natural Breaks	8
Number of licensed physicians per 1,000 population	0.34	0.064	Geometrical interval	5
Number of community health centers per 100,000 population	0.31	0.044	Quantile	4
Number of general practitioners per 10,000 population	0.32	0.030	Geometrical interval	4

#### 3.2.2 Factor detector results

Two healthcare resource factors—total health expenditure per capita and number of licensed physicians per 1,000 population—demonstrated *p*-values greater than 0.05, indicating no statistically significant association with mumps incidence.

As shown in Table 4, the PD values ranged from 0.31 to 0.54. The child dependency ratio (defined as the ratio of population aged 0–14 to the working-age population 15–64) showed the strongest association with mumps incidence (PD = 0.54). This suggests that regions with higher proportions of children relative to working-age adults may experience elevated mumps transmission risks, likely due to increased contact rates among school-aged populations. In comparison, healthcare accessibility factors such as the number of community health centers per 100,000 population and general practitioners per 10,000 population exhibited weaker associations (PD = 0.31 and 0.32, respectively).

#### 3.2.3 Risk detector results

**Figure 4:**
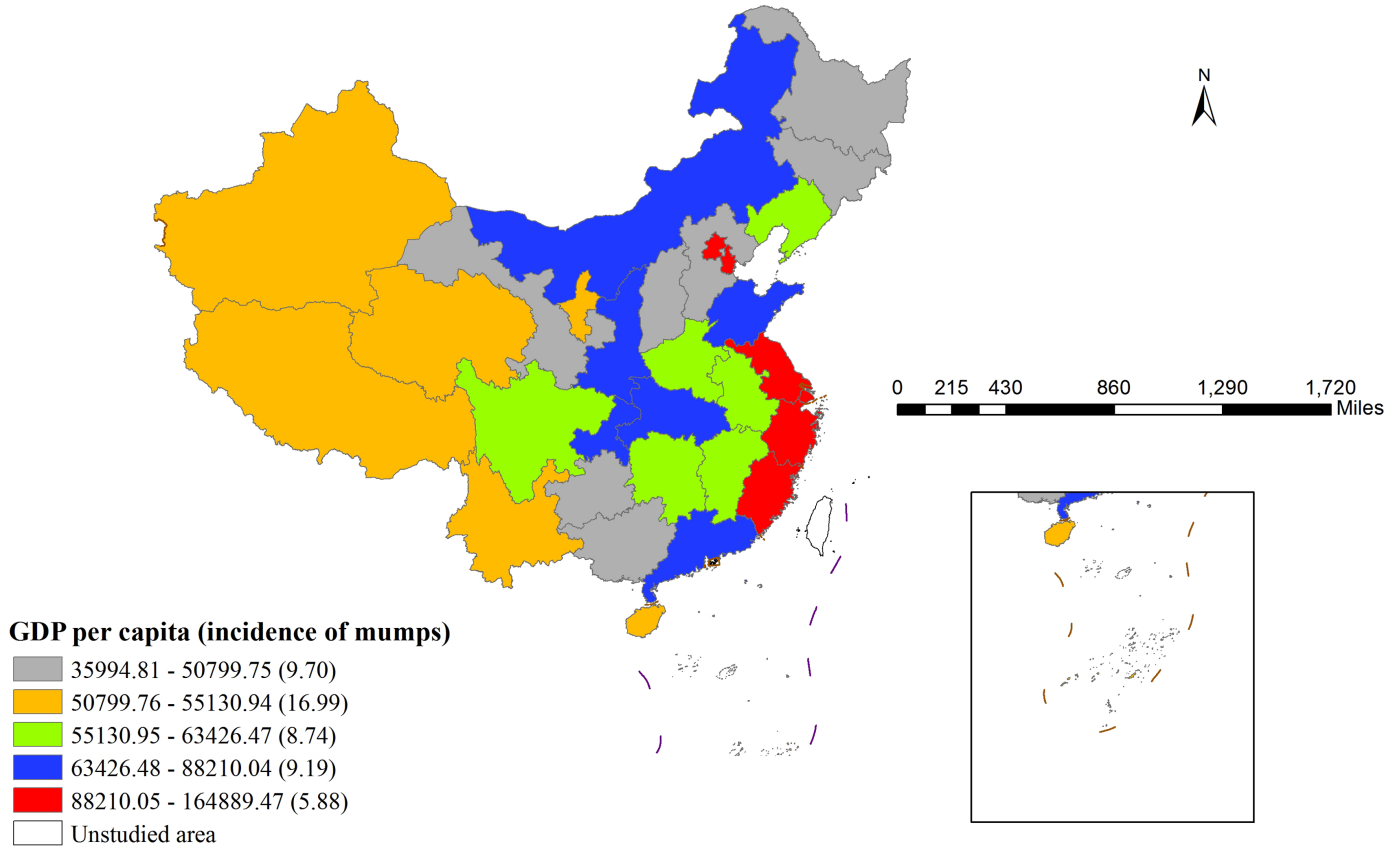
Regional Disparities in GDP per Capita and Associated Mumps Incidence Rates.

The risk detector analysis revealed the average incidence rate of mumps across different stratification levels of each factor, along with an assessment of statistically significant differences between these strata. In this study, we take GDP per capita as an illustrative example. As illustrated in Figure 4, the relationship between GDP per capita and mumps incidence demonstrates a non-linear pattern. The lowest mumps incidence rate (5.88) was observed when GDP per capita ranged between ¥88,210.05 and ¥164,889.47, while the peak incidence (16.99) occurred in the ¥50,799.76–¥55,130.94 GDP per capita range. Notably, the minimum GDP per capita did not correspond to the highest mumps incidence, indicating a complex, non-monotonic relationship between economic development and disease prevalence.

Table 5 presents the significance test results for differences in average mumps incidence rates across different per capita GDP layers (Y indicates significant, N indicates non-significant). The fifth layer corresponds to the highest per capita GDP range (88,210.05 to 164,889.47 RMB) in Figure 3, while the first layer represents the lowest range (35994.81 to 50799.75 RMB). The results demonstrated statistically significant differences in mumps incidence rates between the second layer and all other layers.

**Table 5:** Significance of differences in mean mumps incidence rates across GDP per capita strata.

Stratum	1	2	3	4	5
1					
2	Y				
3	N	Y			
4	N	Y	N		
5	N	Y	Y	N	

Furthermore, the risk detector quantified the association between various factors and mumps incidence by identifying high-risk intervals for each determinant. The peak incidence of mumps corresponds to the high-risk intervals of each contributing factor (23). Therefore, Table 6 presents the identified high-risk intervals for all factors. The identification of these high-risk intervals enables public health authorities to implement more targeted surveillance systems and optimize intervention strategies. By focusing monitoring efforts when factors reach these critical ranges, health systems can enhance early detection and timely response to potential outbreaks. Furthermore, these thresholds serve as valuable reference points for evaluating intervention effectiveness and guiding resource allocation decisions in mumps prevention programs.

**Table 6:** High-risk intervals of factors and their corresponding peak mumps incidence rates.

Factors	High-risk interval	Maximum mumps incidence rate (1/100000)
GDP per capita (RMB)	50799.76–55130.94	16.99
Disposable income per capita (RMB)	20335.11–24562.35	16.00
Urbanization rate (%)	56.54–60.27	14.02
Average household size	2.76–3.19	14.86
Child dependency ratio (%)	28.53–29.52	17.15
PM_2.5_ (ug/m^3^)	10.00–26.26	14.66
Illiteracy rate (%)	5.57–28.09	16.36
Years of schooling (Years)	6.62–9.01	15.28
Number of community health centers per 100,000 population	0.25–0.55	12.69
Number of general practitioners per 10,000 population	2.60–3.26	13.93

**Figure 5:**
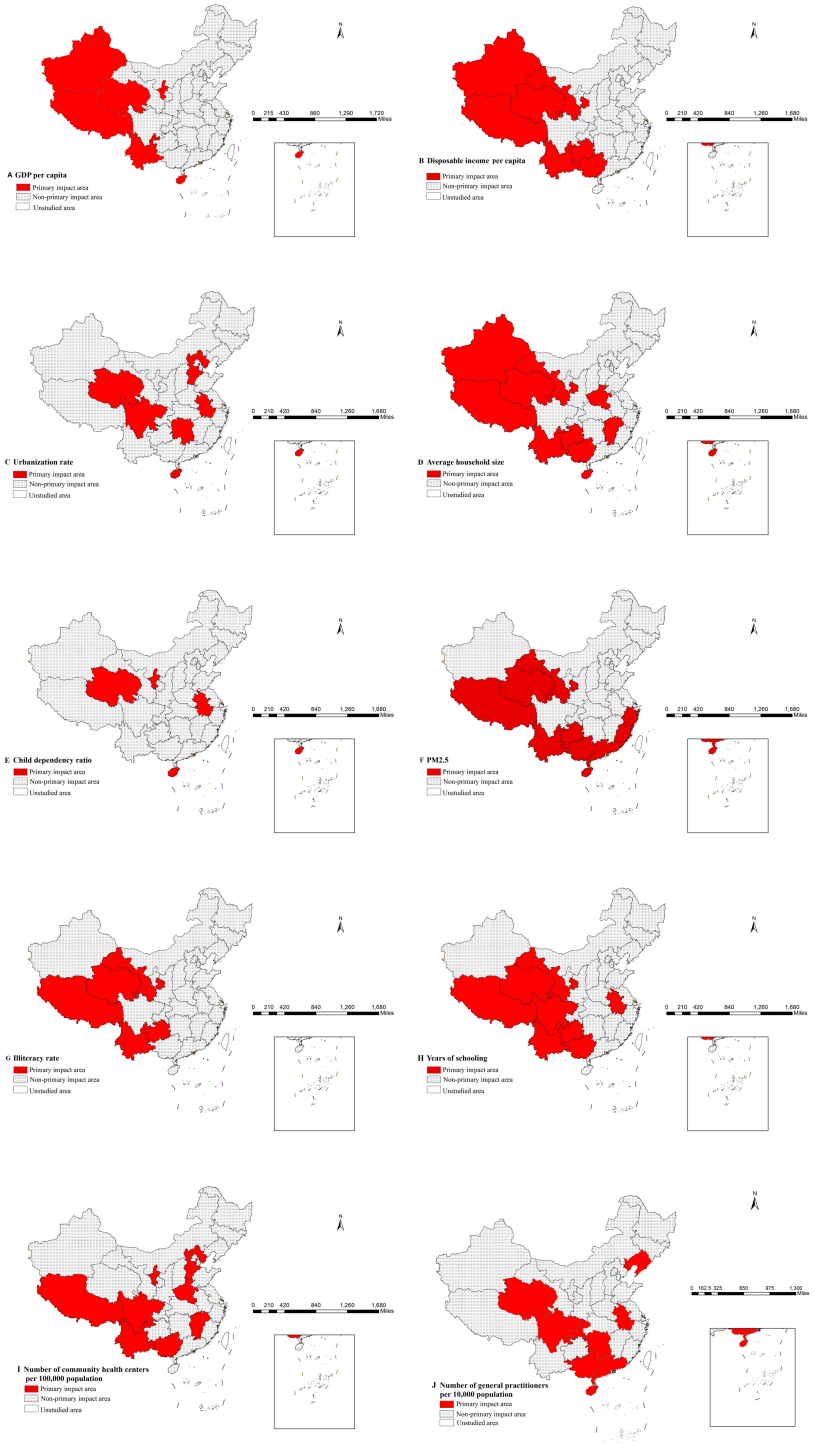
Distribution of main impact area of each factor.

Epidemiologically, these high-incidence zones can be interpreted as primary impact area for each factor (21). These relationships have been visually represented in Figures 5A–J through geospatial visualization techniques. Figures 5A–J systematically illustrates the spatial characteristics of high-incidence zones for each determinant through geospatial visualization. The maps demonstrate the geographic extent of high-risk areas, providing visual context for the quantitative risk intervals identified in Table 6. These representations enable public health practitioners to correlate statistical risk thresholds with specific geographic settings, supporting the development of localized prevention strategies.

#### 3.2.4 Ecological detector results

Our ecological detector analysis revealed important insights into the multifactorial nature of mumps transmission. The comparative analysis of potential differences (PD values) among key determinants (Table 7) showed no statistically significant differences between factor pairs (all *p* > 0.05, denoted by “N”), indicating no single factor demonstrated clear dominance in mumps incidence.

**Table 7:** Significance testing for PD value differences between factors.

Factors	X_1_	X_2_	X_3_	X_4_	X_5_	X_6_	X_7_	X_8_	X_11_	X_12_
X_1_										
X_2_	N									
X_3_	N	N								
X_4_	N	N	N							
X_5_	N	N	N	N						
X_6_	N	N	N	N	N					
X_7_	N	N	N	N	N	N				
X_8_	N	N	N	N	N	N	N			
X_11_	N	N	N	N	N	N	N	N		
X_12_	N	N	N	N	N	N	N	N	N	

This finding carries three key implications: First, the ecological detector results demonstrate that mumps epidemiology is fundamentally shaped by interconnected networks of environmental, social and biological determinants, rather than any single dominant factor. Second, it underscores the need for integrated prevention strategies that simultaneously address environmental, social and biological determinants. Most significantly, this work establishes an urgent research priority: elucidating the precise mechanisms underlying these factor interactions will be essential for designing context-specific control strategies that account for local variations in mumps incidence.

#### 3.2.5 Interaction detector results

Our interaction analysis uncovered meaningful patterns in how various factors jointly influence mumps incidence, with significant implications for public health practice. The consistent enhancement effects observed (all interaction PD values >0.5 in Table 8), particularly the strongest interaction between per capita GDP and illiteracy rate (PD = 0.88), demonstrate that combined factors exert greater influence on disease incidence than their individual effects would affect.

**Table 8:** Interaction effects between risk factors on mumps incidence.

Factors	X_1_	X_2_	X_3_	X_4_	X_5_	X_6_	X_7_	X_8_	X_11_	X_12_
X_1_	0.47									
X_2_	0.73	0.42								
X_3_	0.81	0.57	0.39							
X_4_	0.74	0.6	0.68	0.45						
X_5_	0.86	0.84	0.74	0.84	0.54					
X_6_	0.81	0.73	0.76	0.72	0.79	0.36				
X_7_	0.88	0.7	0.74	0.72	0.87	0.72	0.49			
X_8_	0.8	0.71	0.65	0.69	0.82	0.7	0.81	0.48		
X_11_	0.83	0.75	0.69	0.69	0.79	0.64	0.76	0.65	0.31	
X_12_	0.84	0.69	0.59	0.79	0.74	0.66	0.83	0.64	0.71	0.32

The identified nonlinear enhancement effects among economic indicators and healthcare resources (interactions between x_1_ and x_11_, x_1_ and x_12_) suggest a particularly important dynamic: investments in healthcare infrastructure become increasingly effective at reducing mumps incidence when implemented in conjunction with economic development initiatives. This nonlinear relationship implies that coordinated policy interventions addressing both economic conditions and healthcare access could yield disproportionately large public health benefits compared to isolated improvements in either domain alone.

Similarly, the bivariate enhancement effects observed among other factor pairs reveal synergistic relationships, where simultaneous improvements in two areas produce predictable combined benefits for disease control. These patterns collectively underscore that effective mumps prevention requires integrated public health strategies that account for how different determinants interact within specific contexts.

## 4 Discussion

Our findings revealed significant spatial heterogeneity in mumps incidence across China, with western regions exhibiting persistently higher rates compared to eastern provinces. This pattern may be attributed to multiple interacting factors: (1) disparities in healthcare infrastructure and vaccination program implementation between developed coastal areas and less-developed western regions; (2) variations in population density and contact patterns that facilitate disease transmission; and (3) potential environmental influences such as climate conditions affecting virus survival. The identification of stable high-risk clusters in western China through both LISA and Gi* analyses underscores the need for targeted surveillance and region-specific immunization strategies. These geographical disparities highlighted that uniform national control policies may be insufficient, and precision public health approaches accounting for local epidemiological contexts should be prioritized.

Our analysis of healthcare resource factors—including total health expenditure per capita and the number of practicing physicians per 1,000 population—revealed no statistically significant association with mumps incidence. This finding aligns with the ongoing debate in the literature regarding the role of healthcare resource allocation in infectious disease control. For instance, prior studies suggested that in high-incidence regions, public health funding should prioritize direct interventions such as vaccination coverage rather than merely increasing physician availability or general health expenditures (4, 24).

Notably, the factor detector analysis indicated that the number of general practitioners per 10,000 people and community health centers per 100,000 population had a relatively weak influence (PD values 0.31–0.32), significantly lower than the child dependency ratio (PD value 0.54). This suggested that mumps transmission was more directly affected by factors like population density (8), age structure (e.g., child clustering) (25), and climate conditions (26), whereas healthcare resources (e.g., physician availability) primarily played a role in case management rather than outbreak prevention. Additionally, even with high per capita health spending, limited effectiveness in mumps control may occur if funds are not specifically allocated to vaccination or primary prevention (e.g., health screenings). For example, in some Japanese regions where the mumps vaccine was not part of the national immunization program, targeted local government subsidies still significantly reduced incidence rates (27).

The child dependency ratio exhibited the strongest association with mumps incidence, likely due to increased transmission risks in densely populated pediatric settings, particularly schools and other group environments (10). Surveillance data from Jiangsu Province, China (2023) revealed that 81.75% of mumps cases occurred among students and kindergarten-aged children, with the highest incidence observed in vaccinated children aged 0–12 years (5, 28). Furthermore, children not cohabiting with grandparents demonstrated higher vaccination rates (9). This finding may indirectly influence the association between child dependency ratios and disease incidence, as multigenerational households are more prevalent in regions with higher child dependency ratios.

Risk detector analysis revealed that the lowest per capita GDP stratum did not correspond to the highest mumps incidence. This counterintuitive finding suggests a complex relationship where economic deprivation alone does not predict disease burden. Rather, mid-low income regions appear most vulnerable, likely due to a combination of reduced access to health subsidies (compared to the poorest groups receiving targeted assistance) while still facing resource constraints in healthcare access. Additionally, these regions may experience optimal conditions for disease transmission, including higher population density and mobility patterns that facilitate virus spread, coupled with potentially lower vaccination coverage compared to wealthier areas.

This study quantitatively elucidated the relationships between risk factors and mumps incidence, with the identification of high-risk intervals providing precise intervention targets for disease prevention and control. The synergistic presentation of geospatial visualizations and quantitative data validated the analytical efficacy of the risk detector, establishing a methodological foundation for developing risk factor-based early warning systems.

The interaction detector analysis revealed significant synergistic amplification effects among risk factors, with all pairwise interaction PD values exceeding their independent effects (minimum >0.5). This confirmed that combined environmental and socioeconomic exposed exacerbate disease risk (29). Notably, the interaction between per capita GDP and illiteracy rate demonstrated the most pronounced effect (PD = 0.88), suggesting that regional economic disparities coupled with low education levels may collectively promote mumps transmission by exacerbating inequitable public health resource allocation and health literacy gaps (7, 9). These findings provide quantitative evidence for targeted regional prevention strategies, particularly recommending prioritized interventions in low-GDP/low-education areas through environmental exposure reduction and health education campaigns (30).

To the best of our knowledge, this study represented the first comprehensive application of Geodetector technology to analyze potential influencing factors of mumps at the provincial level in China. The implementation of this innovative methodology not only quantified the individual contributions of various determinants but also, through interaction detector analysis, elucidated the complex synergistic effects among these factors on mumps epidemiology. This approach provided novel insights into the disease transmission mechanisms.

Several limitations should be acknowledged in this study. First, critical variables such as vaccination coverage rates, temperature and humidity were not included due to data availability constraints. Future research should incorporate immunization-related indicators to establish a more comprehensive analytical framework. Second, while our provincial-level analysis revealed important spatial patterns, higher-resolution investigations at municipal or county levels could uncover more detailed geographical heterogeneity in disease distribution and associated risk factors. Addressing these limitations in subsequent studies would enable the development of more precise and effective prevention strategies, ultimately contributing to reduced mumps incidence and transmission nationwide.

## 5 Conclusion

The epidemiological pattern of mumps incidence in China exhibited a distinct west-to-east decreasing gradient and significant spatial clustering. The child dependency ratio emerged as a key determinant of mumps incidence. Based on this finding, we recommend incorporating the child dependency ratio into regional epidemic early-warning systems and prioritizing enhanced vaccination interventions in high-ratio areas to improve disease control efficacy. Furthermore, interaction detector analysis revealed significant synergistic effects among risk factors, with the interaction between per capita GDP and illiteracy rate being particularly pronounced. This suggests that poor economic and educational conditions combined significantly increase mumps transmission risk. These insights provide a critical foundation for targeted public health strategies, underscoring the need for integrated interventions that account for multifactorial synergies to achieve effective mumps prevention and control.

## Data Availability

The original contributions presented in the study are included in the article/Supplementary material, further inquiries can be directed to the corresponding author.

## Footnotes

^1^ https://www.phsciencedata.cn/Share/
